# Evaluation of the Cancer-Preventive Effect of Resveratrol-Loaded Nanoparticles on the Formation and Growth of In Vitro Lung Tumor Spheroids

**DOI:** 10.3390/pharmaceutics16121588

**Published:** 2024-12-12

**Authors:** Elisa A. Torrico Guzmán, Mitchell Gravely, Samantha A. Meenach

**Affiliations:** 1 Department of Chemical Engineering, College of Engineering, University of Rhode Island, Kingston, RI 02881, USA; torrico.elisa@gmail.com (E.A.T.G.);; 2 Department of Biomedical and Pharmaceutical Sciences, College of Pharmacy, University of Rhode Island, Kingston, RI 02881, USA

**Keywords:** resveratrol, nanoparticles, chemoprevention, multicellular spheroids, lung cancer, acetalated dextran

## Abstract

Background: Resveratrol (RSV) is a natural polyphenol that offers antioxidant, anti-inflammatory, and chemopreventive benefits. This project determined the ability of RSV-loaded nanoparticles (NP) to inhibit the growth of lung tumor spheroids in vitro. Methods: RSV was encapsulated in NP comprised of the biodegradable polymer, acetalated dextran. A549 lung cancer cells in two-dimensional and three-dimensional cell culture models were exposed to free RSV and RSV NP to evaluate their effect on cell proliferation and spheroid formation and growth. For prevention studies, spheroids were exposed to free RSV and RSV NP on day 0, and for treatment studies, spheroids were dosed with the same formulations on day 5 after the spheroids had fully formed. Results: The resulting RSV NP were 200 nm in diameter with neutral surface charge and exhibited the ability to control the release of RSV in vitro based on environmental pH. In comparison to free RSV, the RSV NP exerted a greater inhibitory effect on the proliferation and growth of cancer cells and spheroids. Conclusions: RSV NP have the potential to be used as a chemopreventive agent for lung cancer.

## 1. Introduction

Despite advancements in the treatment of cancer, this disease remains an enormous challenge for scientists and clinicians. It is estimated that 1,958,310 new cancer cases will be diagnosed in the U.S. in 2023 and that there will be 609,820 cancer-related deaths [1]. Amongst all cancers, lung and bronchus cancers result in the highest mortality rates (21% of all cancer-related deaths) while accounting for only 12% of anticipated diagnoses. Chemotherapy is a mainstay for the treatment of lung cancer; however, its efficacy is often limited and results in significant side effects. An emerging field in the treatment of cancer is the use of chemopreventive phytochemicals, which are constituents of fruits and vegetables with pharmacological and/or nutritional functions that can prevent oncogenesis [2,3,4,5]. These agents may reverse or inhibit the carcinogenic process at a cellular level, resulting in decreased cancer risk [6,7,8]. The U.S. Food and Drug Administration (FDA) has approved several chemopreventive agents, including tamoxifen [9] and raloxifene for breast cancer risk reduction [10]; celecoxib, to reduce colorectal adenomas in familial adenomatous polyposis [11]; and the human papillomavirus vaccine [12]. Many chemopreventive agents are currently in clinical trials [13,14].

Phytochemicals such as turmeric and green tea can act as blocking agents to prevent DNA mutations, whereas curcumin and gingerol can block molecular pathways that lead to metastasis [15]. Vitamin A, vitamin E, aspirin, selenium, and other chemopreventive agents have been evaluated in clinical trials for the prevention of lung cancer recurrence; however, none of these compounds have shown a clear positive benefit [16]. Compounds such as glucocorticoids and myoinositol have shown promise in preclinical models, but further studies are required to establish their efficacy [17,18]. Overall, there is no FDA-approved formulation available for the prevention of lung tumor formation.

Resveratrol (3,5,4′-trihydroxy-trans-stilbene, RSV) is a phytoalexin that is abundantly found in red grapes, peanuts, and blueberries [19,20]. In 1997, interest in RSV increased when Jang et al. reported its chemopreventive effects in all stages of neoplastic activity, including initiation, promotion, and progression of cell growth [21]. A later study by Dolfini et al. showed that RSV induced drastic growth inhibition in human breast cancer both in vivo and in vitro [22]. While RSV has shown potential as a chemopreventive agent, it exhibits poor water solubility, resulting in limited bioavailability [23]. Approaches to increase the solubility of RSV have included using surfactants and cyclodextrins in formulations [24,25], and nanotechnology has been used to encapsulate and deliver hydrophobic compounds to increase therapeutic bioavailability [26,27,28]. For example, RSV-loaded gel-based nanoparticles (NP) resulted in improved intracellular uptake and cytotoxicity in NCI-H460 lung cancer cells in vitro in comparison to free RSV [29], while RSV-loaded nanostructure lipid carriers exhibited higher penetration into the skin in comparison to solid lipid NP (SLN) [30].

The aim of this project was to evaluate and compare the chemopreventive effect of free RSV and RSV NP on the formation and growth of lung tumor spheroids in vitro. We hypothesized that RSV NP would inhibit or significantly reduce the growth of tumor spheroids more effectively than free RSV both before and after in vitro tumor spheroid formation. RSV-loaded NP was synthesized using the biodegradable polymer acetalated dextran (Ac-Dex) [31], as the facile degradation of Ac-Dex in acidic conditions makes it suitable for the release of therapeutics in the acidic tumor microenvironment [32,33], while its degradation by-products can be released in safe quantities [31].

Both two-dimensional (2D) and three-dimensional (3D) cell culture experiments were conducted using A549 adenocarcinoma lung cancer cells. Monolayer 2D cell culture has been extensively used to test the efficacy of therapeutics in vitro owing to the ease in evaluating drug–cell interactions [34,35]; however, such models do not accurately represent the complex, structural properties of tumors [36,37,38]. A three-dimensional cell culture provides an improved in vitro model for tumors with respect to morphology, response to therapeutics, growth kinetics, presence of a necrotic core, and diffusion limitations [39,40,41]. These characteristics often cause the inner core of tumors to be less accessible to drugs, an effect that is not present in 2D models. As a result, drugs are only able to affect the outer periphery of cells [42,43]. Thus, we evaluated RSV NP for their ability to reduce or inhibit the growth and formation of A549 spheroids in vitro. The novelty of the described formulation lies in the utilization of acid-labile Ac-Dex as an antiproliferative and chemopreventive agent.

## 2. Materials and Methods

### 2.1. Materials

Detailed information on the materials can be found in the Supplemental Information.

### 2.2. Synthesis and Characterization of Acetalated Dextran

Acetalated dextran (Ac-Dex, Figure S1) synthesis and NMR characterization were performed as described previously [44]. The total acetal coverage (conversion of hydroxyl groups) and cyclic acetal coverage were determined by ^1^H-NMR spectroscopy (Bruker 300 MHz NMR, Billerica, MA, USA).

### 2.3. Preparation of Nanoparticles

Resveratrol-loaded nanoparticles (RSV NP) were prepared via a single emulsion and solvent evaporation method. RSV (10 mg) was dissolved in DMSO (1 mL), and 200 μL of the solution was added to 1800 μL of DCM. Ac-Dex (49 mg) was added to 1 mL of the RSV DCM solution to form the organic phase, resulting in 2 wt% initial loading of RSV. PVA (3 *w*/*v*%, 6 mL) in PBS (aqueous phase) was added to the organic phase and sonicated for 60 s with 1 s on/off pulse at an amplitude of 70% (Q500 Sonicator, Qsonica, Newton, CT, USA 100% amplitude = 120 μm). The resulting emulsion was transferred to 40 mL of 0.3% PVA in PBS and stirred for 4 h to allow for organic solvent evaporation and NP stabilization. The final solution was centrifuged at 19,802× *g* and 4 °C for 20 min. The NP was washed twice with basic water, re-dispersed with 0.1% PVA in PBS, frozen overnight, and lyophilized for 24 h. Fluorescent NP was prepared using curcumin (CUR) following the same protocol, adding CUR instead of RSV to the organic phase. Blank NPs were prepared similarly, excluding both RSV and CUR.

### 2.4. Particle Analysis via Scanning Electron Microscopy

The surface morphology and shape of the NP were evaluated by scanning electron microscopy (SEM) with a SIGMA VP Field Emission-SEM (Zeiss, Jena, Germany). A 10 mg/mL suspension of NP in distilled water was placed onto aluminum SEM stubs and dried at room temperature. The samples were sputter coated with a thin film of gold/palladium alloy in a BIO-RAD system at 20 µA for 60 s under argon gas. Images were captured at 5 kV.

### 2.5. Particle Size, Size Distribution, and Zeta Potential Analysis of Nanoparticles

The size, size distribution (polydispersity index, PDI), and zeta (ζ) potential of NP were measured by dynamic light scattering (DLS) using a Malvern Nano Zetasizer (Malvern Instruments, Worcestershire, UK). The NPs were suspended in distilled water (0.5 mg/mL), vortexed, and sonicated for 30 s prior to analysis at 25 °C.

### 2.6. Analysis of Nanoparticle Drug Loading

Drug loading and encapsulation efficacy (EE) of RSV NP and CUR-loaded NP (CUR NP) were determined via ultra-performance liquid chromatography (UPLC, LaChrom, Hitachi, Japan). The detection of RSV was performed using an Ascentis column (C_18_, 5 µm × 150 mm × 4.6 mm) using the following conditions: pump rate of 1 mL/min, absorbance of 306 nm, temperature of 30 °C, 20 μL injection volume, 2 min retention time, and a mobile phase consisting of 60% methanol and 40% aqueous formic acid (0.5 *v*/*v*%). RSV NP samples were dissolved in the mobile phase prior to analysis. The RSV concentration was quantified by comparison with a standard calibration curve of RSV in the mobile phase. CUR loading was determined via fluorescence spectroscopy at 420/520 nm via a Cytation 3 plate reader (BioTek Instruments Inc., Winooski, VT, USA). NP samples were dissolved in DMSO, and CUR concentration was quantified by comparing them with a standard curve of CUR in DMSO. The resulting drug loading and encapsulation efficacy values were calculated using the following equations:

(1) $$\mathrm{Theoretical}\mathrm{Drug}\mathrm{Loading}=\frac{\mathrm{Initial}\mathrm{Mass}\mathrm{of}\mathrm{Drug}}{\mathrm{Mass}\mathrm{of}\mathrm{Drug}+\mathrm{Mass}\mathrm{of}\mathrm{Ac}-\mathrm{Dex}}\times 100\%$$

(2) $$\mathrm{Drug}\mathrm{Loading}=\frac{\mathrm{Mass}\mathrm{of}\mathrm{Drug}\mathrm{in}\mathrm{Nanoparticles}}{\mathrm{Total}\mathrm{Mass}\mathrm{of}\mathrm{Nanoparticles}}$$

(3) $$\mathrm{Encapsulation}\mathrm{Efficiency}\left(\mathrm{wt}\%\right)=\frac{\mathrm{Actual}\mathrm{Drug}\mathrm{Loading}}{\mathrm{Theoretical}\mathrm{Drug}\mathrm{Loading}}\times 100\%$$

### 2.7. In Vitro Release of Resveratrol

The in vitro RSV NP release profiles were determined via a release study of 1 mg/mL of NP suspended in modified phosphate buffer (0.1 M PBS, pH 7.4, 0.5 wt% Tween 80) or modified acetate buffer (0.1 M PBS, pH 5, 0.5 wt% Tween 80). The NP suspensions were incubated at 37 °C and 100 rpm (Digital Heat Block and Orbi shaker, Benchmark Scientific, Edison, NJ, USA). At various time points, NP samples were centrifuged at 23,102× *g* for 5 min at 4 °C to isolate the NPs, and 200 mL of supernatant was withdrawn and replaced by the same amount of fresh buffer. The supernatant samples were analyzed for RSV content by UPLC, as described previously.

### 2.8. In Vitro Cell Culture Conditions

A549 human epithelial adenocarcinoma cells were cultured in DMEM containing 10 *v*/*v*% FBS, 1 mM sodium pyruvate, 1 *v*/*v*% Pen-Strep, and 0.2 *v*/*v*% Fungizone^®^. Cells were incubated at saturated humidity, 37 °C, and 5% CO_2_.

### 2.9. Two-Dimensional Cell Culture and Viability Analysis

The cytotoxic impact of free RSV on 2D A549 cells was evaluated using an acid phosphatase (AP) colorimetric assay. Cells were seeded into flat-bottomed 96-well plates for 24 h (5000 cells/well). Free RSV was dissolved in DMSO to facilitate solubility (0.15 *v*/*v*%), and cells were exposed to free RSV at concentrations up to 500 μM for 48 and 72 h. The AP assay substrate contained 50 mM of pNPP in 1 M of sodium acetate (pH 5) along with Triton X-100 (1 *v*/*v*%) to permeabilize the cell membranes. 20 L of the substrate was added to each well (10 *v*/*v*%) and incubated for 2 h. pNPP is hydrolyzed by the presence of AP in live cells and produces an intense yellow color, which was detected via UV–Vis spectroscopy at 405 nm via a Cytation 3 microplate reader (BioTek Instruments Inc., Winooski, VT, USA).

A 2D viability test using Blank NP (no drug) was performed to evaluate the cytotoxic effect of NPs on A549 cells. As described previously, 24 h after seeding, A549 cells were exposed to up to 12 mg/mL of Blank NP for 24, 48, and 72 h. The viability of the cells was evaluated using a resazurin assay (20 μL/well, 20 mM), and the fluorescence intensity of the samples was detected at 544/590 nm after 4 h of incubation. Relative viability was calculated as the ratio of the absorbance or fluorescence intensity of the sample (cells dosed with free RSV or Blank NP) versus the control (cells with media only), as seen below:

(4) $$\mathrm{Relative}\mathrm{Viability}=\frac{\mathrm{Absorbance}\mathrm{or}\mathrm{Fluorescence}\mathrm{of}\mathrm{Sample}}{\mathrm{Absorbance}\mathrm{or}\mathrm{Fluorescence}\mathrm{of}\mathrm{Control}}$$

### 2.10. Cellular Internalization of Nanoparticles

The ability of NP to be internalized into A549 cells was evaluated using CUR NP to allow for facile imaging owing to the fluorescent nature of CUR. For confocal microscopy, A549 cells were seeded on 35 mm glass bottom petri dishes at 500,000 cells/dish and incubated overnight. The cells were then exposed to 100 μM of CUR NP for 3 h, washed 3× with PBS, stained with CellMask^TM^ deep red plasma membrane stain (0.5 μL/mL in PBS), and fixed with 4% paraformaldehyde in PBS. Images were taken at 100× magnification using a Nikon Ellipse Ti2 inverted confocal fluorescent microscope.

For uptake quantification, A549 cells were seeded in a flat-bottomed 96-well plate for 24 h (5000 cells/well). The cells were exposed to 100 μM of CUR NP for 3 h. After exposure, the particle solution was removed, and cells were washed twice with 200 mM glycine in PBS to remove any unbound particles. The cells were then washed twice with cold PBS and fixed with 200-proof ethanol for 10 min. The internalization of NP internalization was quantified via fluorescence spectroscopy at 420/540 nm using a BioTek Cytation 3 plate reader (Biotek, Winooski, VT, USA).

### 2.11. Formation and Evaluation of 3D Multicellular Spheroids

Multicellular spheroids (MCSs) were formed to evaluate the in vitro effects of free RSV and RSV NP on MCS formation and growth. A549 cells at differing concentrations (3000, 5000, and 8000 cells/well) with 20 μg/mL of collagen type I in complete cell medium were seeded into ultra-low attachment round-bottomed 96-well plates, centrifuged at 1100× *g* for 15 min and incubated for growth observation. Bright-field images of the MCSs were taken using a Cytation 3 imager, and the average size and morphology of the MCSs were evaluated over time using the Cytation 3 software. The presence of live and dead cells in the MCSs at days 1 and 5 of growth was determined using a Live/Dead^®^ assay. The MCSs were stained after gently removing 50 μL of media from the well and replacing the media with 50 μL of Live/Dead^®^ solution, followed by 60 min of incubation prior to fluorescent imaging using the Cytation 3 imager at 494/517 nm and 528/617 nm for calcein AM (green) and ethidium homodimer-1 (red).

### 2.12. Growth Inhibition of Multicellular Spheroids Exposed to Free RSV and RSV NP

The impact of free RSV and RSV NP on the formation and growth of A549 MCSs was evaluated via two types of studies, which were designated as prevention or treatment. MCSs were formed as described previously at 5000 cells/well. For prevention studies, MCSs were exposed to free RSV or RSV NP on the day cells were seeded after centrifugation of the well plates. For treatment studies, MCSs were dosed with free RSV or RSV NP 5 days after MCS seeding and formation. In both the prevention and treatment studies, the plates were incubated at standard conditions, imaged periodically (bright-field), and analyzed for size using a BioTek Cytation 3 imager. The growth of the MCSs was analyzed by calculating the change in size relative to untreated controls, as seen below:

(5) $$∆\mathrm{MCS}\mathrm{Diameter}=\frac{\mathrm{MCS}\mathrm{Diameter}\mathrm{for}\mathrm{Sample}}{\mathrm{MCS}\mathrm{Diameter}\mathrm{for}\mathrm{Control}}$$

### 2.13. Clonogenic Assay Analysis of Multicellular Spheroids

A clonogenic assay was performed on MCSs dosed with free RSV after prevention and treatment studies to evaluate the ability of cells to recover and form colonies. On day 15, MCSs were transferred to a 96-well plate, washed twice with PBS, trypsinized for 5 min to dissociate the MCSs, and pipetted vigorously to break up the MCSs. The resulting cells were seeded in 6-well plates at 500 cells/well for 14 days. The media was then removed from the wells, and the colonies were washed once with PBS and fixed with methanol:acetic acid (3:1) for 20 min. After removing the fixing solution, colonies were stained with 0.5 *w*/*v*% crystal violet in distilled water for 45 min. The wells were washed with distilled water and dried under a hood. Clusters of at least 50 cells were counted as colonies. The survival fraction and plating efficiency were determined using the following equations:

(6) $$\mathrm{Survival}\mathrm{Fraction}=\frac{\mathrm{Number}\mathrm{of}\mathrm{Colonies}\mathrm{in}\mathrm{Sample}\mathrm{Well}}{\mathrm{Number}\mathrm{of}\mathrm{Colonies}\mathrm{in}\mathrm{Control}\mathrm{Well}}\times 100\%$$

(7) $$\mathrm{Plating}\mathrm{Efficiency}=\frac{\mathrm{Number}\mathrm{of}\mathrm{Colonies}\mathrm{in}\mathrm{Well}}{\mathrm{Number}\mathrm{of}\mathrm{Seeded}\mathrm{Cells}}\times 100\%$$

### 2.14. Statistical Analysis

Measurements were performed at least in triplicate, and values are presented as mean ± standard deviation. Half maximal inhibitory concentration (IC_50_) values were calculated using a polynomial regression in PRISM version 5.0 software (GraphPad Software, Inc., San Diego, CA, USA). The statistical significance of the results was determined using Student’s *t*-test. Statistical significance was defined as *p* < 0.05.

## 3. Results

### 3.1. Characterization of Acetalated Dextran

The reaction between dextran and 2-MOP resulted in the synthesis of the biodegradable polymer, acetalated dextran (Ac-Dex) (Figure S1). NMR spectrometry showed a total acetal coverage (conversion of –OH groups) of 80% and cyclic acetal coverage (CAC) of 75% for the Ac-Dex.

### 3.2. Nanoparticle Size, Surface Charge, Morphology, and Drug Loading and Release

**Figure 1:**
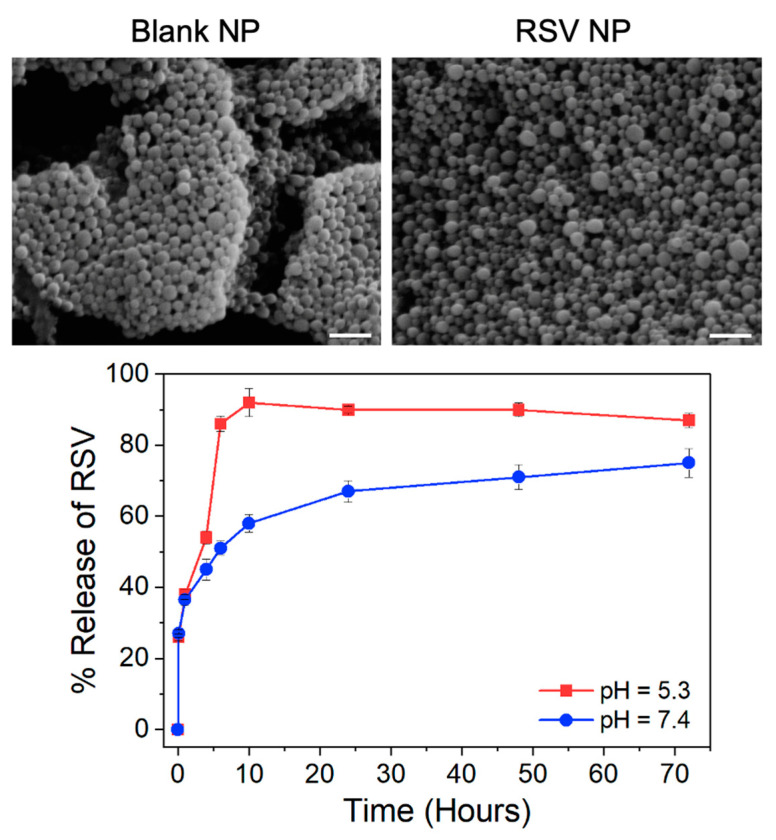
(**Top**) Representative scanning electron images of blank nanoparticles (Blank NP) and resveratrol-loaded nanoparticles (RSV NP). Scale bar = 500 nm. (**Bottom**) In vitro release profiles of resveratrol (RSV) from RSV NP at pH 7.4 and pH 5.3 (*n* = 3).

The diameter, polydispersity index (PDI), and ζ potential (surface charge) of the NP systems were determined via DLS (Table 1). The NP formulations exhibited diameters ≤ 205 nm, with high homogeneity (PDI ≤ 0.15) and neutral surface charges. SEM analysis (Figure 1 Top) revealed smooth, spherical NP and confirmed the narrow size distribution and homogeneity shown via DLS. Furthermore, the imaged NPs were smaller in diameter than what was measured by DLS due to the shrinkage of the particles upon drying [45]. Resveratrol and curcumin were successfully loaded into RSV NP and CUR NP, respectively, and demonstrated similar drug loading and high encapsulation efficiencies (Table 1). The acid sensitivity of the RSV NP formulated with Ac-Dex was shown during the release study (Figure 1 Bottom), where RSV released more slowly at neutral conditions (pH 7.4) in comparison to acidic conditions (pH 5.3), with an initial burst release seen for both pH conditions. The majority of RSV was released from the NP at pH 5.3 (~90% at 6 h), whereas 75% of RSV was released at pH 7.4 after 72 h.

### 3.3. Two-Dimensional Cell Viability upon Exposure to Free Resveratrol and Blank NPs

**Figure 2:**
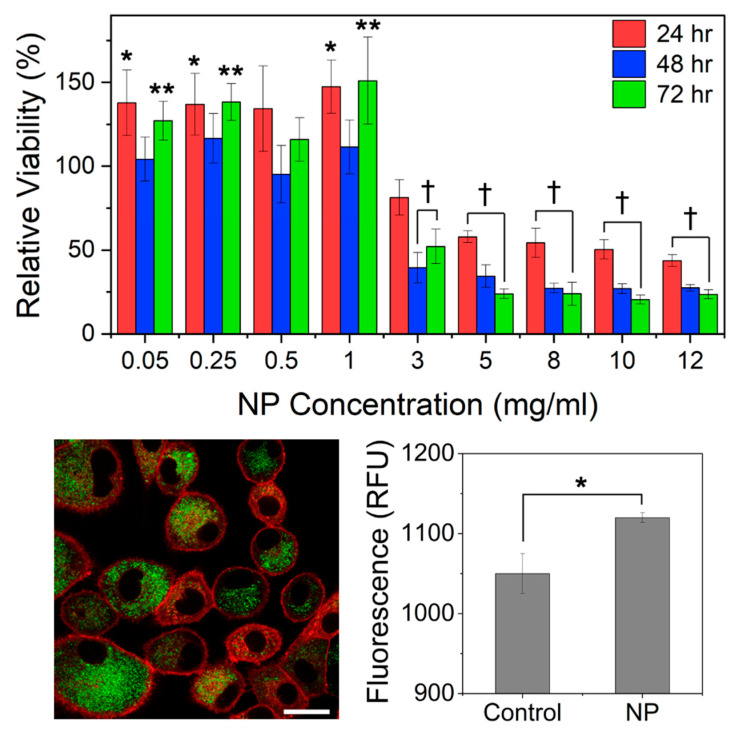
(**Top**) Relative viability of two-dimensional A549 cells exposed to blank nanoparticles at 24, 48, and 72 h; *n* ≥ 3. (**Bottom Left**) Representative fluorescent confocal image of curcumin-loaded nanoparticles (CUR NP) internalized into A549 cells after 3 h; CUR NP = green and cell membrane = red; scale bar = 20 μm. (**Bottom Right**) Fluorescence quantification of CUR NP internalization in A549 cells in relative fluorescence units (RFU) in comparison to a control of no nanoparticles; *n* = 3. * *p* < 0.05, ** *p* < 0.01 represents the comparison to the control with no exposure to NP and † *p* < 0.001 represents the comparison between the marked samples.

When dosed with free RSV, A549 cells exhibited IC_50_ values of 122 and 74 μM after 48 and 72 h of exposure, respectively (Figure S2). For free RSV, the cell culture media contained 0.15 *v*/*v*% DMSO to facilitate RSV solubility, and no negative effects on the viability of the cells from DMSO were observed. As seen in Figure 2 Top, a monolayer of 2D A549 cells exposed to Blank NPs demonstrated a significant reduction in relative viability for NP concentrations ≥ 3 mg/mL, whereas cells exposed to 0.05 to 1 mg/mL of Blank NP exhibited increased viability within the give time exposures.

### 3.4. Cellular Internalization of CUR-Loaded Nanoparticles

As seen in Figure 2 Bottom, after three hours of exposure, CUR NP associated within the cytoplasm of 2D-grown A549 cells and exhibited significantly more fluorescence than the control (cells only), confirming the successful internalization of CUR NP into A549 cells.

### 3.5. Formation, Size Analysis, and Morphology of Multicellular Spheroids (MCSs)

**Figure 3:**
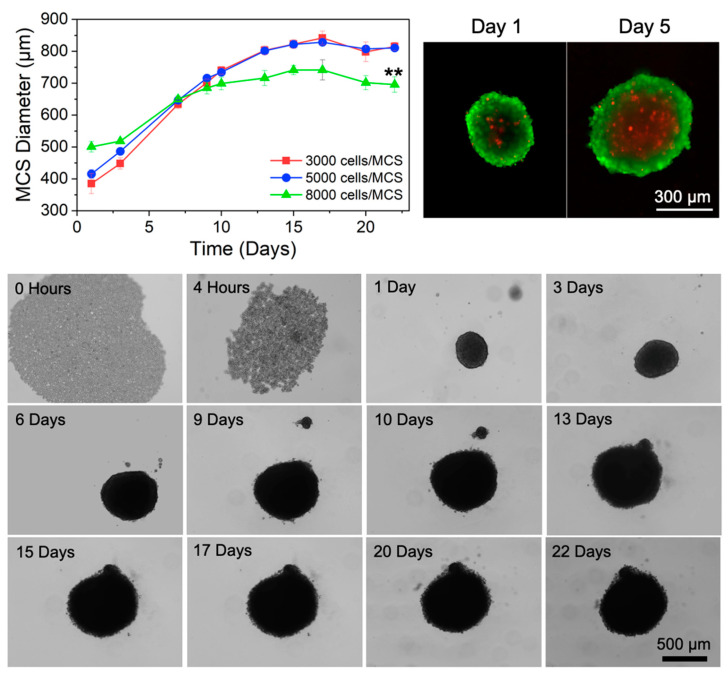
Optimization of A549 multicellular spheroid (MCS) formation. (**Top Left**) Growth analysis of A549 MCSs grown at different initial cell seeding concentrations; ** *p* < 0.01, which represents a comparison of 8000 cells/MCS to 5000 and 3000 cells/MCS on day 22; *n* = 3. (**Top Right**) Representative fluorescent images showing MCS morphology and viability on days 1 and 5, where green = live cells and red = dead cells. (**Bottom**) Representative bright-field images of A549 cells grown at 5000 cells/MCS for 22 days.

Spheroids seeded at 3000 and 5000 cells/MCS exhibited similar growth over the course of the 3D spheroid study (Figure 3 Top Left), whereas spheroids at 8000 cells/MSC were larger on Day 1 but decreased in size starting at Day 9 in comparison to the other two concentrations. The three MCS systems reached their maximum sizes between days 15 and 17, followed by a period where the sizes slightly decreased. The formation and growth of MCSs at 5000 cells/MCS over time is presented in Figure 3 Bottom, where the cells were initially aggregated in a 2D monolayer and formed an organized cluster of cells after four hours. A defined, compact spheroid was present on day 1, and the maximum MCS size at this concentration (828 μm) was reached on day 15 and maintained until day 17. On days 1 and 5, the viability of cells within a representative MCS was evaluated (Figure 3 Top Right), and the resulting fluorescent images show the presence of live cells (green) in the outer region of the MCS and dead cells (red) in the inner core. Furthermore, between days 1 and 5, the MCSs increased in size, and the volume of dead cells increased.

### 3.6. Prevention Study (Growth Inhibition of MCS After RSV Exposure at Day 0)

**Figure 4:**
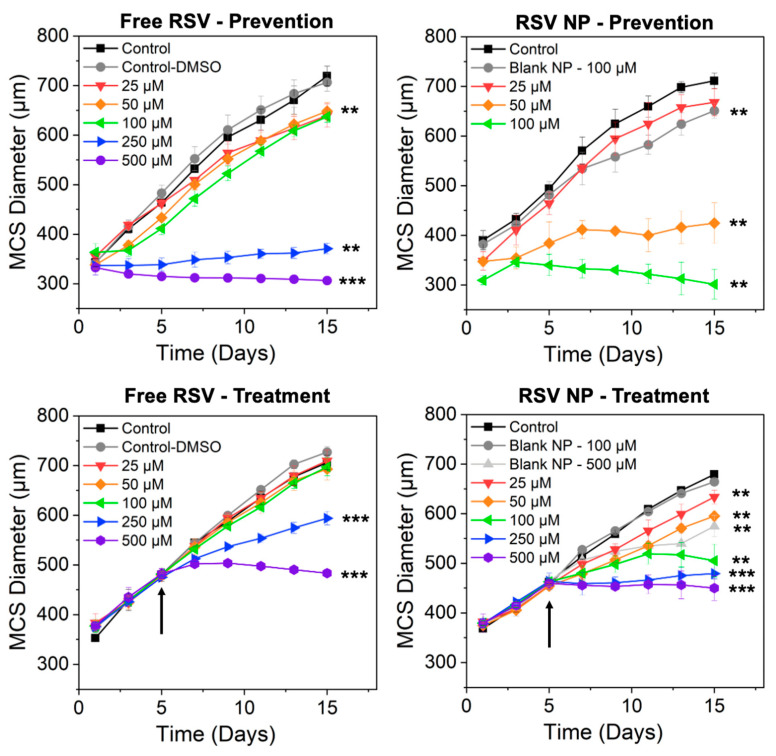
Evaluation of A549 multicellular spheroid (MCS) formation and growth inhibition upon exposure to different resveratrol (RSV) formulations, including free RSV and resveratrol-loaded nanoparticles (RSV NPs). For prevention studies, cells were exposed to the formulations when cells were seeded for MCS formation at day 0, whereas for treatment studies, MCSs were exposed to the RSV formulations 5 days after seeding, as indicated by the black arrows. Control refers to cells not exposed to free RSV or RSV NP (cells only), whereas Control-DMSO refers to cells exposed to media with 0.15 *v*/*v*% DMSO. The concentrations correspond to the amount of RSV cells exposed, regardless of the formulation, whereas the Blank NP formulations correspond to the NP concentration used for the corresponding RSV NP formulation.** *p* < 0.01, *** *p* < 0.001, which represents comparison to the control; *n* ≥ 3.

In the prevention study, MCSs were exposed to varying concentrations of free RSV and RSV NP on Day 0 after the cells were seeded and centrifuged in round bottom well plates. After 15 days, MCSs exposed to 500 μM of free RSV exhibited a reduction in size, whereas MCSs exposed to 250 μM of free RSV showed significant growth inhibition in comparison to the control (Figure 4 Top Left). MCSs exposed to 25, 50, and 100 μM of free RSV showed delayed growth in comparison to the control, and exposure to media with DMSO (Control-DMSO) had no impact on MCS growth. In comparison, exposure to 250 and 500 μM of RSV NP completely prevented the formation of MCSs (e.g., no visible MCSs), whereas exposure to 50 μM of RSV NPs resulted in significant growth inhibition and exposure to 100 μM of RSV NPs resulted in a reduction in the size of MCSs (Figure 4 Top Right). MCSs exposed to 25 μM of RSV NPs did not show a significant difference in size compared to the control, whereas MCSs exposed to the equivalent of 100 μM Blank NPs resulted in an inhibition of MCS growth.

**Figure 5:**
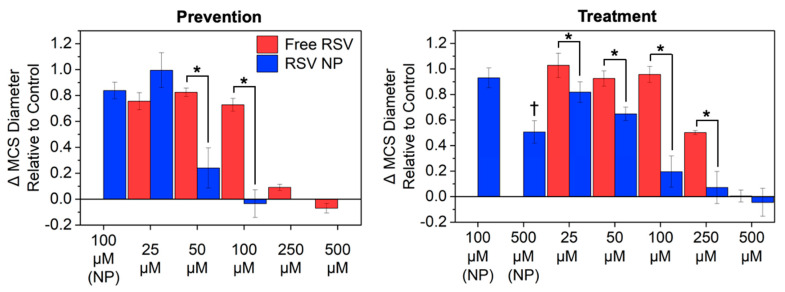
Evaluation of multicellular spheroid (MCS) growth inhibition upon exposure to different resveratrol (RSV) formulations, including free RSV and resveratrol-loaded nanoparticles (RSV NPs). For prevention studies, cells were exposed to the formulations when cells were seeded for MCS formation at day 0, whereas for treatment studies, MCSs were exposed to the RSV formulations 5 days after seeding. The data show the comparison of the change in MCS diameter relative to control between free RSV and RSV NP exposure at day 15. Control refers to cells not exposed to free RSV or RSV NP (cells only), whereas Control-DMSO refers to cells exposed to media with 0.15 *v*/*v*% DMSO. The concentrations correspond to the amount of RSV cells exposed, regardless of the formulation, whereas the Blank NP formulations correspond to the NP concentration used for the RSV NP formulation. * *p* < 0.05, which represents a comparison between free RSV and RSV NPs and † *p* < 0.05 in comparison to control; *n* ≥ 3.

The change in spheroid diameter relative to the control on day 15 of the prevention study was evaluated to demonstrate the impact of RSV NPs in comparison to free RSV on MCS growth inhibition, as seen in Figure 5 Left. MCSs exposed to Blank NPs and 25 μM RSV exhibited no significant inhibition of growth in comparison to control, whereas MSC growth was significantly inhibited upon exposure to 50 and 100 μM of RSV NPs in comparison to the same concentrations of free RSV. MCSs exposed to 250 and 500 μM of free RSV exhibited growth inhibition or a reduction in size, whereas exposure to 250 and 500 μM of RSV NPs resulted in no MCS formation.

**Figure 6:**
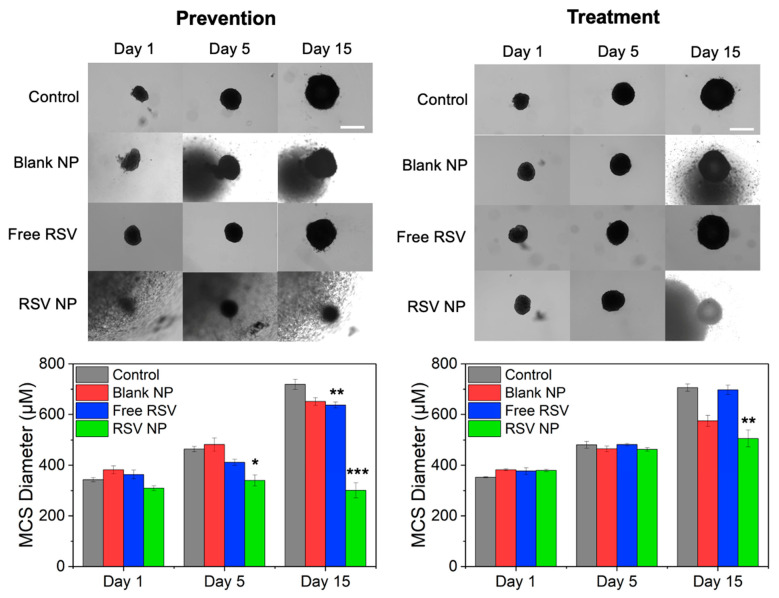
Comparison of A549 multicellular spheroid (MCS) growth upon exposure to different resveratrol (RSV) formulations, including free RSV and resveratrol-loaded nanoparticles (RSV NP). For prevention studies, cells were exposed to the formulations when cells were seeded for MCS formation at day 0, whereas for treatment studies, MCSs were exposed to the RSV formulations 5 days after seeding. The data include (**Top**) representative bright-field images and (**Bottom**) corresponding quantification of MCS growth upon exposure to 100 µM of free RSV, RSV NPs, and Blank NPs equivalent to 100 µM RSV NP by mass. Control MCSs were exposed to media only. Scale bar = 500 µm. * *p* < 0.05, ** *p* < 0.01, *** *p* < 0.001, which represents a comparison to the control for each time point; *n* ≥ 3.

Representative bright-field images of MCSs dosed with 100 μM RSV formulations and quantitative data in comparison to the control for days 1, 5, and 15 are shown in Figure 6 Left to compare MCS growth inhibition and show spheroid morphology over time between the RSV formulations (free RSV versus NP RSV). Exposure to both free RSV and RSV NP resulted in spheroid size reduction after days 5 and 15, with RSV NP being significantly more effective than free RSV at reducing MCS size.

### 3.7. Treatment Study (Growth Inhibition of MCSs After RSV Exposure at Day 5)

In the treatment study, MCSs were exposed to varying concentrations of free RSV and RSV NPs on day 5 rather than day 0, as was seen in the prevention study. As seen in Figure 4, Bottom Left, the MCSs grew similarly up to day 5 prior to exposure to the RSV formulations. On day 15, no difference was observed between MCSs exposed to lower concentrations of free RSV (≤100 μM RSV) in comparison to the control, whereas MCSs exposed to 250 μM of free RSV exhibited an inhibition in growth and those exposed to 500 μM experienced a reduction in size. MCSs exposed to RSV NPs experienced significant spheroid growth inhibition for concentrations ranging from 25 to 500 μM in comparison to the control, whereas MCSs exposed to Blank NPs exhibited no significant inhibition of growth, and a significant inhibition in growth was observed for MCSs exposed to Blank NPs at 500 μM (Figure 4 Bottom Right).

As seen in Figure 5 Right, for the treatment study, a significant inhibition in MCS growth was observed for MCSs exposed to RSV NPs in comparison to free RSV for RSV concentrations ranging from 25 to 250 μM. Growth was completely inhibited in MCSs exposed to both 500 μM free RSV and RSV NPs. Representative bright-field images of MCSs dosed with 100 μM RSV formulations (Figure 6 Right) show how the MCSs decreased in size when exposed to RSV NPs but not free RSV.

### 3.8. Clonogenic Assay

**Figure 7:**
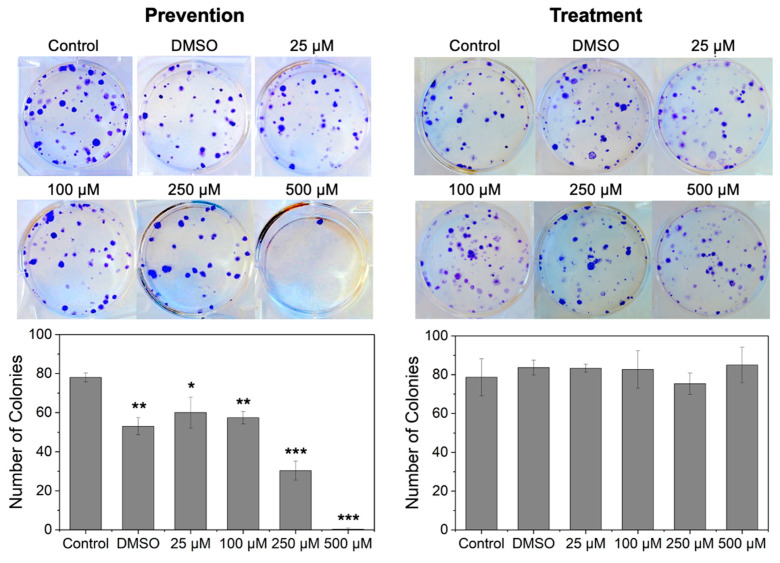
Clonogenic assay analysis of A549 multicellular spheroids (MCSs) upon exposure to free resveratrol (RSV). For prevention studies, cells were exposed to free RSV when cells were seeded for MCS formation, whereas for treatment studies, MCSs were exposed to free RSV 5 days after seeding. The data include (**Top**) representative images of colony formation and (**Bottom**) quantification of colony formation after 14 days. DMSO refers to MCS-exposed media with 0.15 *v*/*v*% DMSO. Data are presented as the number of colonies compared with the untreated control (mean ± standard deviation, *n* = 3). * *p* < 0.05, ** *p* < 0.01, *** *p* < 0.001, which represents a comparison to the control (no exposure to RSV).

As seen in Figure 7, cells from MCSs exposed to free RSV exhibited differing behavior in colony formation for the prevention and treatment studies. In the prevention study, the formation of colonies followed a similar pattern as seen in the inhibition of the growth of MCSs, where an increase in RSV concentration resulted in an increase in the inhibition of colony formation. In contrast, for the treatment study, cells from the MCSs treated with free RSV formed a similar number of colonies, irrespective of RSV concentration, in comparison to the control.

## 4. Discussion

The properties of the biodegradable Ac-Dex are important in order to obtain favorable NP formulations. During Ac-Dex synthesis, dextran transitions from a more hydrophilic compound to a hydrophobic one due to the conversion of –OH groups to acetals on the backbone of its structure. High acetal coverage ensures the stability of PVA-coated NP in aqueous solution [46,47], and high –OH conversion and cyclic acetal coverage allow Ac-Dex to exhibit pH-tunable hydrolysis-driven degradation, where the polymer degrades in acidic conditions more readily than neutral conditions [48]. Overall, the acetal coverage ensures that the Ac-Dex is appropriate to use in the proposed NP formulation.

In order to ensure their efficacy in drug delivery applications, NPs need to exhibit appropriate size, homogeneity, and surface charge characteristics [49]. The resulting RSV NP exhibited diameters of ~200 nm and were homogeneous in size, which meets the requirements for their use as drug delivery vehicles. Furthermore, while neutral and slightly polar molecules have the tendency to agglomerate in aqueous environments, the RSV NP formulations were well dispersed in water due to their PVA coating, as PVA is known to improve colloidal stability and reduce the agglomeration of NP due to its steric effect [50]. The RSV release studied demonstrated the acid-sensitivity of Ac-Dex, where the RSV NPs degraded faster in acidic conditions than in neutral ones. This can potentially be used for the fast release of RSV in acidic conditions such as those seen in tumors or inflammation [32], which is applicable to the proposed use of these NPs.

Since 2D cell culture has been used extensively for preliminary drug screening, we determined the in vitro therapeutic concentrations of free RSV using a two-dimensional (2D) monolayer of A549 lung adenocarcinoma cells prior to translation into a more complex three-dimensional (3D) system. Initially, the IC_50_ values were analyzed to determine the appropriate RSV dosage concentrations for the in vitro 3D MCS studies, where cells were exposed to RSV concentrations both below and above the 2D IC_50_ values (e.g., 25 to 500 μM). While Blank NP resulted in low cell viability at higher concentrations, at lower NP concentrations, the viability was shown to increase during the given time exposures, which likely indicates that the dextran released during NP degradation may have been used as a nutrient by the cells [51].

In preliminary 2D cell uptake studies, RSV and curcumin (CUR) were co-encapsulated in NPs to utilize the fluorescence of CUR and the therapeutic nature of RSV collectively; however, a decrease in cell numbers was noticed after one hour of exposure to the CUR-RSV NP formulation. As a result, a CUR NP formulation was used for the 2D cell internalization studies. CUR is a small molecular weight compound similar to RSV, and the CUR NP exhibited a similar size and surface charge to RSV NP (Table 1), demonstrating their viable use in these studies. Previous studies have shown that NP 100–200 nm in diameter can readily be internalized into cells [52,53], and the RSV NP and CUR NP formulations were within this optimal size range. The accumulation of NPs within the cells, as shown in this study, can allow for RSV to be released directly into the cells following NP internalization and exert its therapeutic effect rather than having to internalize on its own accord.

The use of 3D MCSs offers advantages over 2D in vitro cell culture as MCSs offer a better representation of the structure of in vivo tumors and better mimic drug–cell interactions [54,55,56]. Thus, 3D lung cancer spheroids were formed using A549 cells and were optimized for in vitro growth by evaluating the impact of the initial cell seeding concentration on spheroid growth. A cell seeding concentration of 5000 cells/MCS was determined to be optimal based on our studies and was used for subsequent studies for the free RSV and RSV MCS NPs exposure studies. Since the MCSs at this concentration maintained a maximum size at day 15, future 3D studies were concluded at this time point.

In vitro MCSs larger than 200 μm may display a heterogeneous structure with a peripheral proliferating region, an intermediate quiescent region, and an inner necrotic core due to limited oxygen and nutrient transport [57]. Larger in vitro spheroids (>500 µm) often present a similar physicochemical gradient to that seen in micrometastasis and 0.5–1 mm^3^ avascular tumors [58]. In this study, in vitro MCSs developed morphology similar to in vivo tumors with respect to a proliferating outer surface and core with dead cells. These findings are consistent with other studies that demonstrate similar features in MCSs after 4 days [59]. Overall, these studies show the importance of using a more physiologically relevant cell culture platform for the evaluation of the chemopreventive potential of cancer therapeutics.

In order to determine the therapeutic potential of RSV NPs in the inhibition of spheroid growth, preventive and treatment studies were completed comparing the impact of free RSV and RSV NP on in vitro MCSs. For prevention studies, cells were exposed to the RSV formulations when cells were initially seeded for MCS formation on day 0, whereas for treatment studies, MCSs were exposed to the RSV formulations 5 days after seeding. In both studies, exposure to 500 μM RSV resulted in a significant inhibition or prevention of MCS growth, which was enhanced by the NP carrier. In addition to the impact of the RSV, it is likely that the NP physically interfered with the aggregation of cells during MCS formation in the prevention study, thereby enhancing the inhibitive effect of the RSV NP formulation.

Exposure to 50 and 100 μM RSV NP enhanced the efficacy in inhibiting spheroid growth in comparison to free RSV for both preventive and treatment studies. In previous studies, higher concentrations of free RSV (>100 μM) resulted in mitochondrial dysfunction and promoted apoptosis in human hepatoblastoma cells [60], and with RSV solid lipid NPs that were used as an effective neuroprotectant in glioma cells [61]. Recent studies have shown several potential mechanisms related to the antiproliferative impact of RSV on lung cancer cells, including the binding of RSV to the Akt protein and destruction of the intracellular antioxidant pool in cells, in addition to the activation of the NGFR-AMPK-mTOR pathway [62,63,64,65]. The polymer effect on formulation safety was previously evaluated in a PLGA system, where a concentration of 50 μM of RSV-loaded PLGA NP was found to be beneficial as an antioxidant in cochlear cells and remained under the cytotoxic limits of NP [65]. The RSV-loaded Ac-Dex NP system developed in this project agreed with these findings. Overall, these findings imply an enhanced preventive effect of RSV in an NP formulation in comparison to free RSV, where a significantly lower concentration of RSV NP was needed to achieve spheroid growth inhibition via chemoprevention. Furthermore, after RSV treatment, spheroid growth was inhibited for all RSV NP concentrations. This outcome demonstrates another potential application of RSV in an NP formulation for the treatment of solid tumors.

Cells from MCSs dosed with free RSV in the prevention and treatment studies were harvested and reseeded to determine their recovery and potential to form colonies. The resulting data showed that while exposure to RSV in the treatment study had a significant impact on the formation of colonies at a variety of RSV concentrations, no impact was seen on colony formation for cells disassociated in MCSs from the treatment study. These findings suggest that prevention chemotherapeutic treatment using RSV NP might be advantageous for reduced cancer recurrence in comparison to the direct treatment of already formed tumors.

Given the complexity of lung cancer with respect to the heterogeneity of the disease, we would like to point out that the use of a single lung adenocarcinoma cell type is one of the major limitations of this study. Furthermore, the utilization of single-cell MCSs may not adequately represent the cellular complexity of clinical tumors, which could limit their utility in translational cancer research [66,67]. Future studies that would enhance the current project include looking at different lung adenocarcinoma cells and/or the incorporation of different cell types (e.g., white blood cells) into the 3D system, as has been described in reviews elsewhere [68,69].

## 5. Conclusions

We developed a resveratrol-loaded nanoparticle formulation based on the biodegradable polymer Ac-Dex, resulting in spherical RSV NP that exhibited homogeneous dispersion, smooth surfaces, and appropriate diameters for cellular uptake. The evaluation of RSV NP in the inhibition of A549 tumor spheroid growth showed that this chemopreventive agent results in an enhanced therapeutic effect as a nanoparticle formulation when compared to free RSV. High concentrations of RSV NP inhibited the formation of MCSs, while lower concentrations significantly reduced the growth of tumor spheroids. These results were consistent in both the prevention and treatment studies. These findings represent potential alternative uses for RSV NPs in the chemoprevention and treatment of lung cancer tumors.

## Figures and Tables

**Table 1:** Characteristics of resveratrol (RSV)-loaded nanoparticles (RSV NP), blank NP without RSV (Blank NP), and curcumin (CUR)-loaded NP (CUR NP), including their diameter, polydispersity index (PDI), zeta (ζ) potential, drug loading, and encapsulation efficiency (EE) (mean ± standard deviation, *n* = 3).

System	Diameter (nm)	PDI	ζ Potential (mV)	Drug Loading (μg drug/mg NP)	EE (%)
RSV NP	205 ± 6	0.12 ± 0.04	−2.9 ± 0.4	14.6 ± 2.3	73.2 ± 11.6
Blank NP	192 ± 3	0.12 ± 0.02	−5.6 ± 1.3	N/A	N/A
CUR NP	176 ± 8	0.15 ± 0.03	−4.1 ± 0.5	14.7 ± 0.1	73.7 ± 0.1

## Data Availability

Data is contained within the article or Supplementary Materials.

## Supplementary Materials

The following supporting information can be downloaded at https://www.mdpi.com/article/10.3390/pharmaceutics16121588/s1, Figure S1: Chemical structures of (Top) trans-resveratrol and (Bottom) synthesis of acetalated dextran (Ac-Dex) from parent dextran. PPTS = pyridinium p-toluenesulfonate (reaction catalyst), DMSO = dimethylsulfoxide (solvent); Figure S2: Cell viability analysis using an acid phosphatase assay. The relative viability of A549 cells exposed to free resveratrol (RSV) was evaluated using the following conditions and times: (Top) 2D monolayer after 48 h and (Bottom) 2D monolayer after 72 h; Table S1: Clonogenic assay data representing the number of colonies, plating efficiency (%), and survival fraction (%) of A549 cells from multicellular spheroids exposed to free resveratrol (RSV) in prevention and treatment studies. For prevention studies, cells were exposed to free RSV when cells were seeded for MCS formation, whereas for treatment studies, MCS were exposed to free RSV 5 days after seeding. Control-D refers to cells exposed to media with 0.15 *v*/*v* % DMSO. Data is presented as number of colonies compared with untreated control (mean ± standard deviation, *n* = 3). Statistical analysis was performed using Student’s *t*-test, where * *p* < 0.05, ** *p* < 0.01, *** *p* < 0.001, which represents comparison to the control (no exposure to RSV).

## Abbreviations

Ac-Dex = acetalated dextran; CAC = cyclic acetal coverage; CUR = curcumin; DAPI = 4′,6-diamidino-2-phenylindole; DCM = dichloromethane; DLS = dynamic light scattering; DMEM = Dulbecco’s modified Eagle’s medium; DMSO = dimethyl sulfoxide; EE = encapsulation efficacy; FBS = fetal bovine serum; GFP = green fluorescent protein; MCSs = multicellular tumor spheroids; 2-MOP = 2-methoxypropene; NP = nanoparticles; PBS = phosphate buffered saline; PDI = polydispersity index; pNPP = p-nitrophenyl phosphate; PPTS = pyridinium p-toluenesulfonate; PVA = polyvinyl alcohol; RSV = resveratrol; TEA = triethylamine.
