# Supplementary material for: Evaluation of the Cancer-Preventive Effect of Resveratrol-Loaded Nanoparticles on the Formation and Growth of In Vitro Lung Tumor Spheroids
Source: Pharmaceutics. 2024 Dec 12;16(12):1588. doi: 10.3390/pharmaceutics16121588 (PMC11677875; doi:10.3390/pharmaceutics16121588)
Supplement: Supplementary file 1 [file pharmaceutics-16-01588-s001.zip › pharmaceutics-3244883-supplementary.pdf]

pharmaceutics

Article

Supplementary Materials: Evaluation of the Cancer-Preventive Effect of Resveratrol-Loaded Nanoparticles on the Formation and Growth of In Vitro Lung Tumor Spheroids

Elisa A. Torrico Guzmán, Mitchell Gravely and Samantha A. Meenach

Materials

Dextran from Leuconostic mesenteroides (MW 9,000 - 11,000), pyridinium p-toluenesulfonate (PPTS, 98%), anhydrous dimethyl sulfoxide (DMSO, ≥ 99.9%), 2-methoxypropene (2-MOP, 97%), triethylamine (TEA, ≥ 99%), deuterium chloride (DCl, 35 wt% in D_2O, 99% atom D), dichloromethane (DCM, > 99.8%), anhydrous sodium acetate, Triton™ X-100, Tween® 80, methanol (HPLC grade, ≥ 99.9%), crystal violet, and acetic acid (≥ 99%) were obtained from Sigma-Aldrich (Natick, MA, USA). Deuterium oxide (D_2O, 99.8% atom D), polyvinyl alcohol (PVA, 88% hydrolyzed, average MW 22,000), resazurin (sodium salt), and curcumin (CUR, > 98%) were obtained from Acros Organics (Geel, Belgium). Formic acid (FA, ≥ 99.5%) and glycine (≥ 98.5%) were obtained from Fisher Scientific (Somerville, NJ, USA). Phosphate buffered saline 10X (PBS) was obtained from Research Products International (RPI) (Mount Prospect, IL, USA). Resveratrol (RSV, > 98%) was purchased from AdipoGen™ (Sunnyvale, CA). A549 human lung adenocarcinoma epithelial cells were obtained from American Type Culture Collection (ATCC, Manassas, VA, USA). Fetal bovine serum (FBS) was obtained from Atlanta Biologics (Flowery Branch, GA, USA). Collagen rat tail type I was obtained from Corning Inc. (Corning, NY, USA). Dulbecco's Modified Eagle's Medium (DMEM, 4.5 g/L d-glucose and L-glutamine), Trypsin-EDTA (0.25%), phenol red, calcein AM, and ethidium homodimer-1 were obtained from Life Technologies™ (Carlsbad, CA, USA). Sodium pyruvate, Pen-Strep (100 U/ml penicillin, 100 μg/ml streptomycin), and Fungizone® (0.5 μg/ml amphotericin B, 0.41 μg/ml sodium deoxycholate) were obtained from GE Health Life Sciences (Pittsburgh, PA, USA). p-nitrophenyl phosphate disodium salt hexahydrate (pNPP) was purchased from MP Biomedicals (Illkirch, France). Crystal violet in powder was obtained from Beantown Chemical (Hudson, NH, USA). CellMask™ deep red plasma membrane stain was obtained from Invitrogen (Carlsbad, CA, USA). Paraformaldehyde (32%, formaldehyde, aqueous solution) was purchased from Electron Microscopy Sciences (Hatfield, PA, USA).

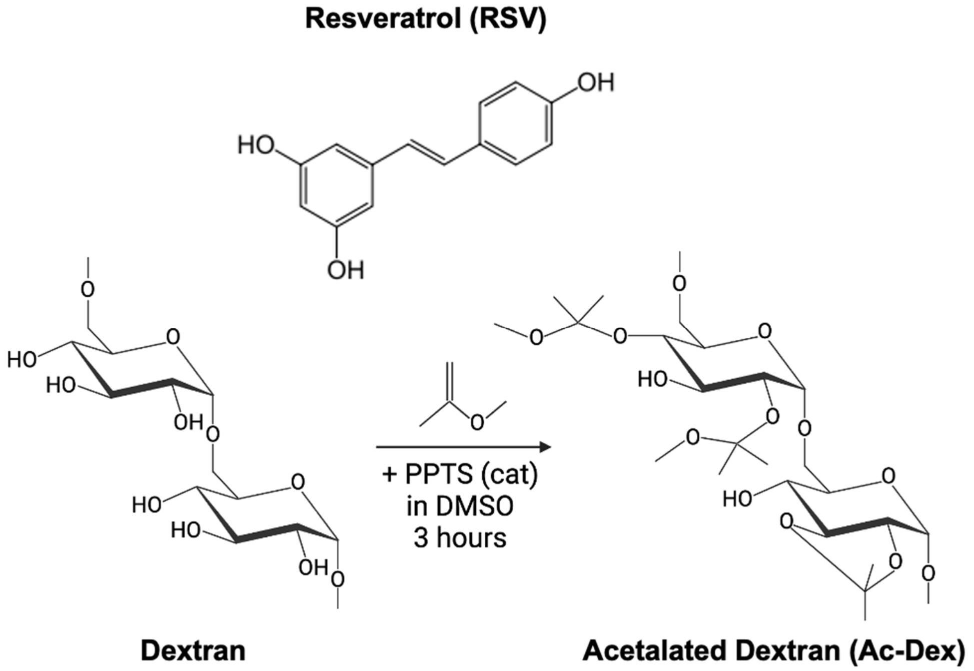

Figure S1. Chemical structures of (Top) trans-resveratrol and (Bottom) synthesis of acetalated dextran (Ac-Dex) from parent dextran. PPTS = pyridinium p-toluenesulfonate (reaction catalyst), DMSO = dimethylsulfoxide (solvent).

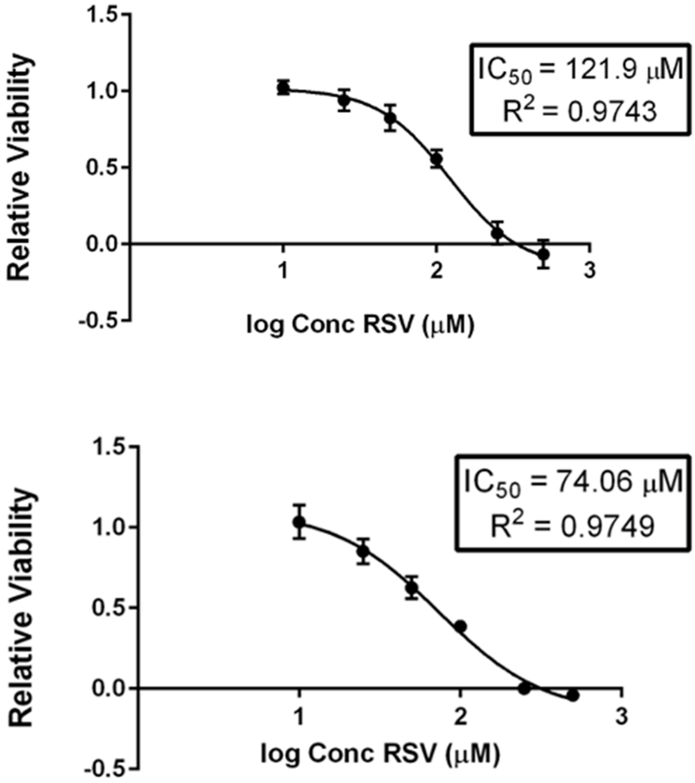

Figure S2. Cell viability analysis using an acid phosphatase assay. The relative viability of A549 cells exposed to free resveratrol (RSV) was evaluated using the following conditions and times: (Top) 2D monolayer after 48 hours and (Bottom) 2D monolayer after 72 hours.

Table S1. Clonogenic assay data representing the number of colonies, plating efficiency (%), and survival fraction (%) of A549 cells from multicellular spheroids exposed to free resveratrol (RSV) in prevention and treatment studies. For prevention studies, cells were exposed to free RSV when cells were seeded for MCS formation, whereas for treatment studies, MCS were exposed to free RSV 5 days after seeding. Control-D refers to cells exposed to media with 0.15 v/v % DMSO. Data is presented as number of colonies compared with untreated control (mean ± standard deviation, n = 3). Statistical analysis was performed using Student's *t*-test, where $^{**}p < 0.01$, $^{***}p < 0.001$, which represents comparison to the control (no exposure to RSV).

			**Treatment**			
**Group**	**Number of colonies**	***p*-value**	**Plating efficiency (%)**	***p*-value**	**Survival fraction (%)**	**p-value**
Control	78 ± 2	N/A	15 ± 1	N/A	100 ± 3	N/A
Control-D	53 ± 4 **	0.002	11 ± 1 **	0.002	68 ± 6 **	0.002
25 μM	60 ± 8	0.048	12 ± 2	0.048	77 ± 10	0.048
100 μM	57 ± 3 ***	0.001	12 ± 1 ***	0.001	74 ± 4 ***	0.001
250 μM	30 ± 5 ***	0.001	6 ± 1 ***	0.001	39 ± 6 ***	0.001
500 μM	0.3 ± 0.6 ***	0.000	0.1 ± 0.1 ***	0.000	0.4 ± 0.7 ***	0.000
			**Prevention**			
**Group**	**Number of colonies**	***p*-value**	**Plating efficiency (%)**	***p*-value**	**Survival fraction (%)**	**p-value**
Control	79 ± 10	N/A	16 ± 2	N/A	100 ± 12	N/A

Control-D	84 ± 4	0.472	17 ± 1	0.472	107 ± 5	0.472
25 μM	83 ± 2	0.491	17 ± 1	0.491	106 ± 3	0.491
100 μM	83 ± 10	0.639	17 ± 2	0.639	105 ± 12	0.639
250 μM	75 ± 6	0.636	15 ± 1	0.636	96 ± 7	0.636
500 μM	85 ± 9	0.455	17 ± 2	0.455	108 ± 12	0.455
